# Erythromycin as an alternative to reduce interfering extra-cardiac activity in myocardial perfusion imaging

**Published:** 2010-06

**Authors:** Mariza Vorster, MM Sathekge, P Rheeder

**Affiliations:** Department Pharmacology, Steve Biko Academic Hospital, University of Pretoria, South Africa; Department of Nuclear Medicine, Steve Biko Academic Hospital, University of Pretoria, South Africa; Department of Nuclear Medicine, Steve Biko Academic Hospital, University of Pretoria, South Africa; Department Clinical Epidemiology, Steve Biko Academic Hospital, University of Pretoria, South Africa

**Keywords:** erythromyacin, myocardial perfusion imaging, artefacts, improving diagnostic accuracy

## Abstract

**Objectives:**

We sought to determine whether taking oral erythromycin prior to SPECT myocardial perfusion imaging with Tc99m-sestamibi would reduce the amount of interfering extra-cardiac activity and improve the image quality.

**Methods:**

A total of 96 patients who were routinely referred for myocardial perfusion imaging were randomly assigned to one of two groups. Patients in group A received 500 mg of non-enterically coated erythromycin orally one hour prior to image acquisition (45 patients). Patients in group B received diluted lemon juice which comprises the current standard of care in our department (51 patients). A two-day protocol was followed and study participants received the same intervention on both days. Planar images of both the stress and rest images were assessed visually by three experienced nuclear medicine physicians for the presence of interfering extra-cardiac activity. Physicians were blinded to the detail of the protocol and independently assessed the images.

**Results:**

The qualitative results favoured lemon juice in reducing the amount of interfering extra-cardiac activity. The overall incidence of interfering extra-cardiac activity was 46.15% in the lemon juice group vs 55.56% in the erythromycin group. However, this difference was not found to be statistically significant (*p* = 0.36). The use of a MYO:EXT ratio similar to the one described by Peace and Lloyd,^11^ appeared promising in quantifying interfering extra-cardiac activity.

**Conclusion:**

The difference between the effect of erythromycin and lemon juice on interfering extra-cardiac activity appears statistically insignificant and erythromycin could therefore be considered as a suitable alternative to lemon juice.

## Summary

Single-photon emission computed tomography (SPECT) myocardial perfusion imaging is a means of providing functional information about the left ventricle and myocardial perfusion.

Accurate and reliable determination of left ventricular function and perfusion are important to guide therapeutic management, improve risk stratification, and provide prognostic information in the cardiac evaluation of patients. Hence it is of great importance that the results are reliable and reproducible. However, artifacts related to abdominal activity frequently affect the accuracy. This is due to the fact that the two most commonly used radiopharmaceuticals: Tc99m methoxy isobutyl isonitrile (sestamibi) and Tc-99m tetrofosmin are both excreted by the hepatobilliary system.^1^,^2^

Due to the fact that the heart lies on the diaphragm just above the left lobe of the liver and in the vicinity of the bowel, attenuation and scattered radioactivity from these organs can interfere with both visual and quantitative interpretation and cause artifacts during reconstruction of SPECT images, especially in the inferior and infero-septal walls of the left ventricle. This is most commonly associated with rest or pharmacological stress studies in which the hepatic uptake is most marked, and it is also seen more frequently in women.^3^

Since the introduction of sestamibi, it has been recommended that a fatty meal be given to reduce interference from liver and gall bladder activity.^4^ The recent procedure guidelines adopted by the British Cardiac Society, however, state that the value of a fatty meal is uncertain and may be counter-productive if there is gastro–duodenal reflux, or if the tracer reaches the transverse colon.^5^

Various proposals have been made to reduce extra-cardiac activity and its unwanted effects, with mixed results. Some of these include prone imaging, pixel truncation, ECG-gated acquisition and supplemental exercise with pharmacological stress.^1^,^5^,^6^ Pharmacological interventions have included the use of cholecystokinin and metoclopramide. Recently, the effect of lemon juice has also been evaluated.^7^-^9^ The latter comprises the current protocol in our department.

To our knowledge, erythromycin has not been used in a study to evaluate the effect on interfering extra-cardiac activity. The rationale behind the use of erythromycin is the following. Erythromycin has been shown to increase gastric emptying, possibly by mimicking the effect of motilin after binding to the antral and duodenal motilin receptors. This leads to strong phase 3 contractions of the interdigestive motor complex.^7^ In addition, some studies have suggested that erythromycin stimulates gall bladder motility.^10^

In our study, we sought to determine whether taking oral erythromycin prior to SPECT myocardial perfusion imaging with Tc99m-sestamibi would reduce the amount of interfering extra-cardiac activity and improve image quality and patient management. An additional aim was to validate the recently described method to quantify the level of interfering extracardiac activity – the MYO:EXT ratio.^11^

## Methods

Study participants were patients who were routinely referred for myocardial perfusion imaging for known or suspected coronary artery disease at the Nuclear Medicine Department of the Steve Biko Academic Hospital (formerly known as the Pretoria Academic Hospital). Patients were evaluated for possible exclusion criteria, after which written consent was obtained from all study participants. See Table 1 for inclusion and exclusion criteria. A list of possible interactions with erythromycin was made available to all study participants (Table 2).

**Table 1.** Inclusion And Exclusion Criteria

*Inclusion criteria*	*Exclusion criteria*
• Patients who were routinely referred for myocardial perfusion imaging for known or suspected coronary artery disease at the Nuclear Medicine department of the Steve Biko Academic Hospital. (Previously known as the Pretoria Academic Hospital.	• Pregnancy
	• Patients younger than 18 years of age
	• Known hypersentivity to erythromycin
	• Patients using gastric motility agents which could potentially cause the gallbladder to contract
	• Previous billiary or gastrointestinal surgery
	• Hepatic impairment
	• Significant history of upper gastro-intestinal complaints
	• Patients using essential medication with known erythromycin interactions.

**Table 2.** Possible Drug Interactions With Erythromycin

• Digoxin	• Tacrolimus
• Sildenafil	• Cyclosporine
• Dispoyramide	• Lovastatin or simvastatin
• Warfarin	• Bromocriptine
• Theophylline	• Cilostazol
• Alprazolam or triazolam	• Quinidine
• Ergotamine	• Vinblastine
• Carbamaepine	• Other antibiotics

**Fig. 1.**
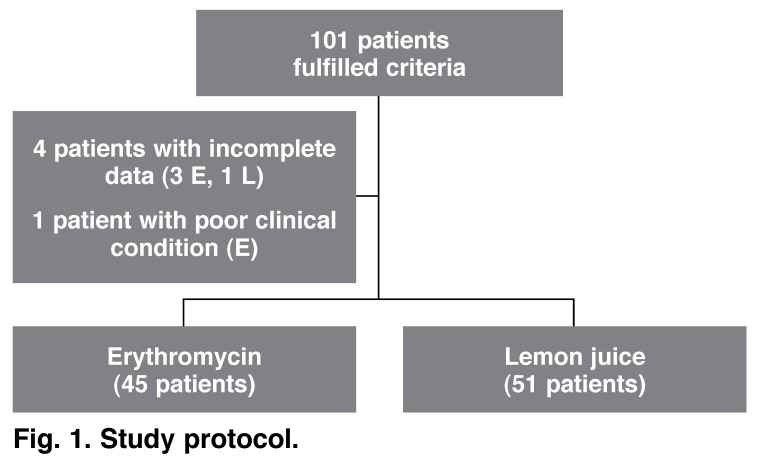
Study protocol.

The study commenced following ethics approval and 101 patients were enrolled in the study between 19 May and 22 September 2008. Four patients were excluded from the study due to incomplete data and one was unable to complete the study due to severe nausea and hypotension (Fig. 1). Clinical characteristics of patients in both groups are listed in Table 3.

**Table 3.** Clinical Characteristics Of Patients In Each Group

	*Erythromycin (n = 45)*	*Lemon juice (n = 51)*	p*-value*
Age (mean)	59.04	58.10	0.631
Gender (male/female)	33/12	35/16	0.738
Caucasian	34	35	0.345
Black	6	9	
Indian	5	4	
Coloured	0	3	
Co-morbidities			
Acute coronary syndrome	40	45	0.849
Systemic hypertension	36	40	0.984
Diabetes mellitus	10	12	0.833
Dyslipidaemia	31	19	0.006
CVS failure	10	7	0.300

Both the allocation of patients as well as the administration of the interventions was administered by the nursing staff of the department. All physicians were blinded to the detail of the protocol. Patient preparation consisted of the following: at least a four-hour fast prior to stress testing (usually overnight), no caffeine for at least 24 hours and discontinuation of β-blockers, calcium channel blockers, nitrates and any other drugs considered necessary. Patients were verbally checked for abstinence from these substances prior to the study.

**Fig. 2.**
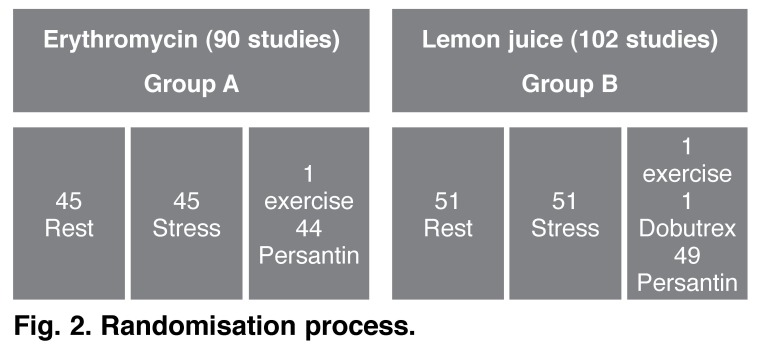
Randomisation process.

Patients who were allocated to group A received 500 mg of non-enterically coated erythromycin orally one hour prior to image acquisition.^7^,^12^ Where such patients required aminophylline, the dose was reduced to 75 mg intravenously (IV) (erythromycin metabolite increases the plasma level of aminophylline by 35%).^13^ Patients allocated to group B received 250 ml of diluted lemon juice (150 ml lemon juice + 100 ml water) 10 min after tracer injection, which is the standard of care in our department (as validated by Cherng *et al*.^9^) Fig. 2 shows the randomisation process.

A two-day protocol was performed with IV injection of 555 MBq of Tc99m-sestamibi on both days and study participants underwent mostly pharmacological stress testing (0.56 mg/kg dipyridamole IV) combined with low-level exercise. Stress images were acquired 30 min after tracer injection and rest images between 45 and 60 min following tracer injection.

## Image acquisition and processing

**Fig. 3.**
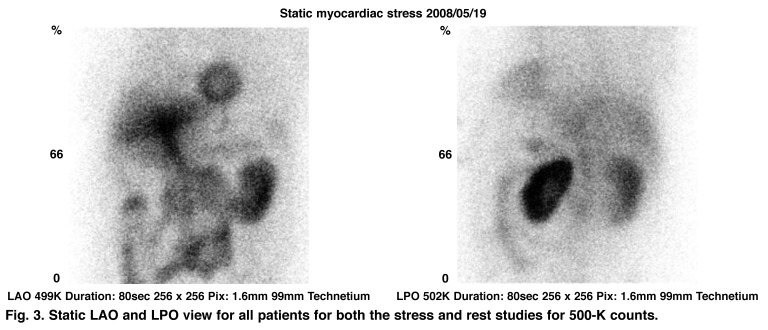
Static LAO and LPO view for all patients for both the stress and rest studies for 500-K counts.

Patients were imaged supine using a double-headed Siemens E-CAM gamma camera with both detectors configured at 90˚ and fitted with low-energy, high-resolution collimators. Gated SPECT imaging was performed using eight frames. Thirty projection images, each of 25 seconds’ duration, were acquired on a 64 × 64 matrix over a 90˚ counter-clockwise rotation with a starting angle of 45˚ (step-and-shoot mode). A zoom of 1.45 was applied resulting in a pixel size of 1.6 mm. A Butterworth filter was applied (order 5, cut-off 0.4) and filtered back projection was used for reconstruction. In addition, a static LAO and LPO view was acquired for all patients for both the stress and rest studies for 500-K counts (Fig. 3)

## Statistical analysis

Sample size was determined based on similar studies published recently. Pearson’s Chi-squared test was used to determine whether the baseline characteristics of the two groups were comparable. With the exception of dyslipidaemia, no statistically significant differences were found. A two-sample *t*-test with equal variances was used to determine the correlation between observer evaluation and the MYO:EXT ratio. In addition, the kappa statistic was calculated (using STATA 10) to evaluate inter-observer agreement for more than two observers with two possible outcomes (yes/no). A *p*-value of less than 0.05 was considered statistically significant in all calculations.

## Data analysis

Three experienced nuclear medicine physicians evaluated static images (LAO and LPO views) of both the stress and rest studies of all study participants for the presence or absence of interfering extra-cardiac activity. Observers evaluated the images independently of one another and were blinded to the clinical information as well as the protocol details.

**Fig. 4.**
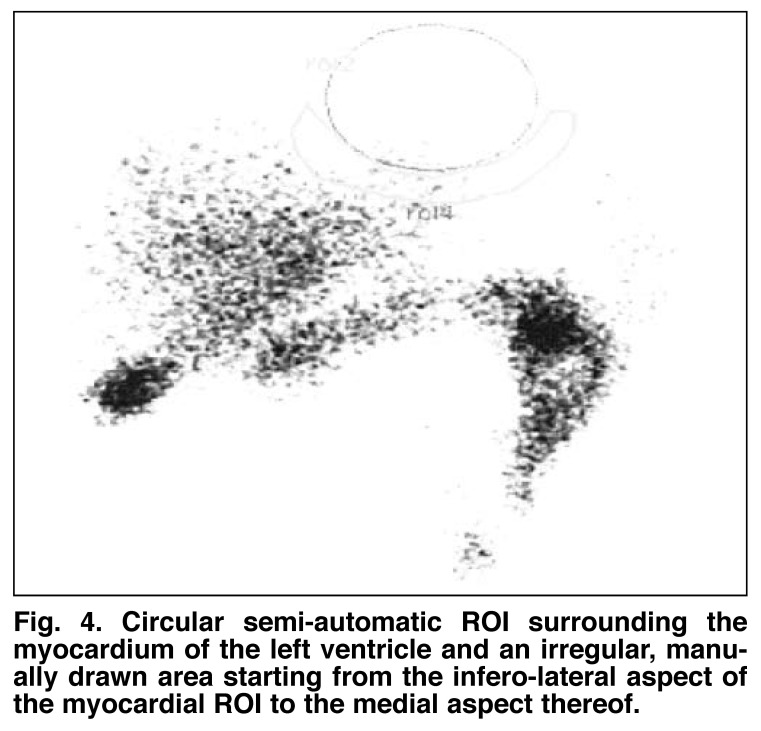
Circular semi-automatic ROI surrounding the myocardium of the left ventricle and an irregular, manually drawn area starting from the infero-lateral aspect of the myocardial ROI to the medial aspect thereof.

The raw anterior data for both parts of each study were selected and regions of interest (ROIs) were created as follows: a circular semi-automatic ROI surrounding the myocardium of the left ventricle and an irregular, manually drawn area starting from the infero-lateral aspect of the myocardial ROI to the medial aspect thereof (Fig. 4). ROIs were copied between stress and rest studies of individual patients to increase reproducibility.

The mean counts per pixel were obtained for both ROIs and were used to calculate the ratio between myocardial activity (MYO) and extra-cardiac activity (EXT) for each patient in a manner similar to the one described by Peace and Lloyd.^11^ The aim was to validate the use of the MYO:EXT ratio in quantification of interfering extra-cardiac activity in our patient population.

## Results

The patient characteristics (Table 3) did not demonstrate any statistically significant differences between the two study groups in terms of demographics or co-morbidity (*p* > 0.05) with the exception of dyslipidaemia, which was more prevalent in the erythromycin group

**Fig. 5.**
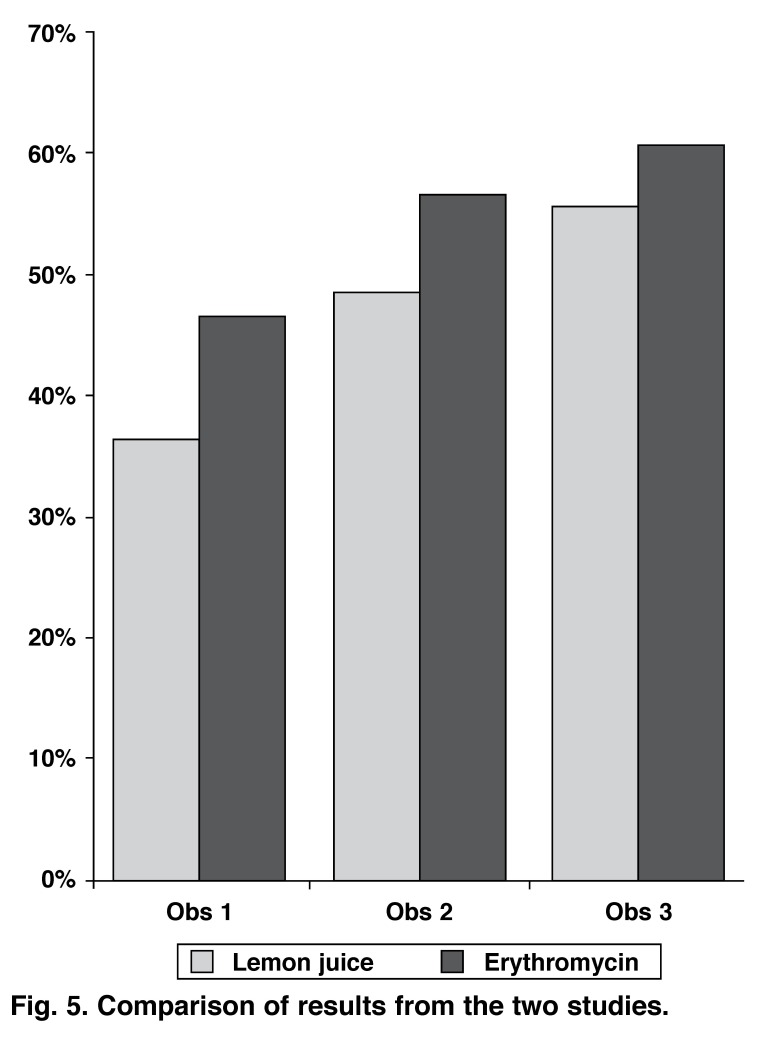
Comparison of results from the two studies.

All three physicians determined that the frequency of interfering extra-cardiac activity was higher in the erythromycin study group when compared with the lemon juice group. Differences between the two groups varied between 6 and 10% among the various observers. Interference was consistently judged to be higher on the stress studies when compared to the rest studies (Fig. 5, Table 4).

**Table 4.** Quantitative Results

*Interfering extra-cardiac activity*	*Erythromycin*	*Lemon juice*
Observer 1	42/90 (46.67%)	38/102 (37.25%)
Stress/rest	21/21	21/17
Observer 2	51/90 (56.67%)	50/102 (49.02%)
Stress/rest	28/23	28/22
Observer 3	55/90 (61.11%)	57/102 (55.88%)
Stress/rest	29/26	29/28

For the purpose of validating the MYO:EXT ratio, the absence or presence of interfering activity was considered to be present when at least two or three physicians concurred. The kappa statistic revealed fair to moderate inter-observer agreement, with better agreement noted on the rest studies (κ = 0.42) when compared to the stress studies (κ = 0.37). These values were calculated independently of the intervention that was used.

The presence of interfering extra-cardiac activity was consistently judged higher in the erythromycin group (55.56%) when compared to the lemon juice group (46.15%) (*p* = 0.36). A strong and statistically significant relationship was found with the qualitative evaluation when using the rest MYO:EXT (*p* = 0.0002). Using the MYO:EXT ratio generated from the stress images did not yield a statistically significant result (*p* = 0.0568). Table 5 combines the results of all three observers and evaluates the stress and rest studies separately. The presence or absence of interfering activity was considered to be positive when at least two or three physicians concurred.

**Table 5.** Results Of Stress And Rest Studies Separately

	*Stress*		*Rest*	
	*Y*	*N*	*Y*	*N*
Number	57/96	39/96	48/96	48/96
Myo:Ext	1.12	1.22	1.08	1.24
± SD	0.26	0.22	0.21	0.19
*p*-value (difference between Y and N)	0.057		0.0002	
95% CI	1.05-1.19	1.14-1.29	1.02-1.14	1.18-1.29

Myo:Ext = myocardium-to-extra-cardiac activity ratio; Y = presence of interfering extra-cardiac activity; N = absence of interfering extracardiac activity; CI = confidence interval.

A sub-group of 48 patients was selected from the study participants in whom an additional MYO:EXT ratio was calculated on the lateral images of both the stress and rest images. An average value was generated for each of the 48 patients using both the anterior and lateral images. This was calculated separately for stress and rest studies and correlated with the qualitative results. The results were similar to those provided in the table above with only the ratios obtained from the rest images proving to be statistically significant (*p* = 0.0002).

## Discussion

SPECT myocardial perfusion imaging with sestamibi is a widely used, non-invasive diagnostic method for assessing patients with coronary artery disease with regard to severity, extent, prognosis and therapeutic response. The initial myocardial uptake of sestamibi is related to regional myocardial blood flow and is dependent on a mitochondrial-derived membrane electrochemical gradient. Sestamibi is predominantly excreted by the hepatobilliary system. Unfortunately, this mechanism of excretion is frequently the source of artifacts due to abdominal activity, which affects both observer interpretation and reconstruction. Various proposals have been made to overcome this problem in order to improve diagnostic accuracy.

Recently, a prospective, randomised trial with 86 patients and 82 controls was conducted to evaluate the use of metoclopramide. This study followed two previous studies with conflicting results. Therefore the aim was to conclusively determine whether metoclopramide was useful in reducing artifacts related to abdominal activity in myocardial perfusion SPECT. In this study metoclopramide showed neither a qualitative nor quantitative impact on abdominal activity in myocardial perfusion imaging.^8^

The use of metoclopramide is based on the rationale that it is a prokinetic drug, which should enhance gastric clearance of Tc99m MIBI. However, a recent study failed to demonstrate any effect of metoclopramide on interfering extra-cardiac activity.

A study done recently by Cherng et al.^9^ concluded that diluted lemon juice accelerated the transit of tetrofosmin through the liver parenchyma, leading to improved image quality on myocardial perfusion SPECT images. Prior to this, various studies had been done in order to evaluate the effects of water, milk, milkshakes, and fatty meals on imaging time.^11^ The rationale behind these protocols is the following:

• Clearance of activity located in the gall bladder and hepatic duct into the duodenum and bowel may be stimulated by fatty meals, milkshakes and intravenous cholecystokinin.
• Water and sandwiches have been used to fill the stomach and push the bowel away from the myocardium, thus exerting a volume effect. In addition, peristaltic gastric motility may be stimulated by oral consumption following pre-injection fasting, and the upper gastro-intestinal tract activity may therefore be diluted.^6^

A study by Peace and Lloyd^11^ evaluated the effect of imaging time, the radiopharmaceutical used, and full-fat milk or water on interfering extra-cardiac activity in myocardial SPECT in 260 patient acquisitions. The following conclusions were made from this study:

• The quantitative results indicated that oral administration of full-fat milk or water made no significant difference to extracardiac activity.
• There was no significant difference between tetrofosmin and sestamibi at any point.
• Delayed imaging significantly reduced extra-cardiac activity interfering with observer interpretation.
• More importantly, however, was the development of a method to quantify the myocardial-to-extra-cardiac activity ratio, which correlated significantly with observer interpretation of myocardial perfusion studies.^11^

## Other studies performed

Boz *et al.*^4^ investigated the volume effect of the stomach in patients undergoing a same-day exercise–rest protocol. After the rest study, patients were given either 200 ml of water and a sandwich or were fasted and imaged again within 30 mins. Quantitative and observer analysis showed significantly reduced activity inferior to the left ventricle in the meal group due to an expanded stomach.^4^

Hurwitz *et al*.^6^ studied the effect of a milkshake taken either immediately after injection or just before imaging. An early drink decreased gallbladder activity, but had no effect on the liver parenchyma at 15 and 110 min. Patients drinking a milkshake just prior to imaging showed a prompt 26% reduction of activity inferior to the myocardium.^6^

Van Dongen and van Rijk qualitatively investigated the effects of administering 450 ml of water 10 mins before imaging and/or 150 ml of whole milk 10 mins after injection. Visual analysis showed significantly fewer patients with interfering activity when a milk-only protocol was used, compared with water only or no drink, for images reconstructed by FBP. A combined milk-and-water protocol showed the least number of studies with interfering activity, compared with water-only and milk-only protocols.^1^

Most recently, Hara *et al*.^3^ reported on the use of soda water (given in the form of a carbonated soft drink) in conjunction with adenosine stress myocardial perfusion imaging and in the Monzen position. (This is a body-positioning manoeuvre to increase the distance between the heart and the intestine by raising the left arm and curving the trunk slightly to the right.) According to their report, a carbonated beverage distended the stomach with gas and lessened artifacts from intestinal radiotracer activity. Additional benefits included the faster clearance of gas and a smaller amount of fluid needed to distend the stomach, which is especially desirable in patients with left ventricular dysfunction.^3^

Interfering extra-cardiac activity therefore remains a significant problem and a limitation to accurate interpretation of myocardial perfusion imaging. In our study, interfering extra-cardiac activity was present overall in 54% of the stress images and 48% of the rest images, when the results were combined from all three observers for both groups. Almost all the stress studies were performed with Persantin, confirming once again the higher incidence of interfering activity on pharmacological stress studies.

In our Nuclear Medicine Department, the use of diluted lemon juice has replaced the former protocol of bread and milk. Lemon juice was perceived to reduce the amount of interfering activity significantly, although the effects of the two protocols were never formally compared. When evaluating the qualitative results of this study, lemon juice appeared to be superior in reducing the amount of interfering activity, with all three observers reporting less interference in the lemon juice group. However, this was not found to be statistically significant. The superiority of lemon juice in this setting might be explained solely on the basis of a much larger volume of fluid consumed, which might have led to stomach distension.

## Limitations of this study

The experimental design was sub-optimal, with individuals being exposed to the same intervention during both parts of the study (stress and rest). A better design might have been to expose individuals to both interventions on different days. However, the inherent differences in interfering activity on stress and rest studies (even in the absence of any intervention) present a diagnostic dilemma.

A third and even a fourth comparison group would have been helpful in validating the results with one group serving as a control (receiving no intervention) and the other receiving milk and bread (according to our previous protocol). However, considering the decrease in the amount of interfering activity perceived with the use of lemon juice, the addition of the latter two groups were considered to be unethical.

Upon final statistical analysis, the study appeared to be slightly underpowered, which could have resulted in a type II error. There were suspected errors during the randomisation process, which might have resulted in the higher incidence of dyslipidaemia in the erythromycin group. The calculation method of the MYO:EXT ratio as described by Peace and Lloyd^11^ could not be replicated exactly due to differences in acquisition parameters as well as the software used for image processing.

Despite the above limitations, the use of diluted lemon juice appeared to be beneficial in reducing interfering extra-cardiac activity in MPI studies where sestamibi was used. Furthermore, erythromycin should be considered as an alternative to lemon juice where patients find it more acceptable and in particular when taking into account the low cost and the low incidence of adverse effects.

Our secondary aim was to validate the method as described by Peace and Lloyd^11^ to quantify the amount of interfering extracardiac activity (MYO:EXT). A Xeleris workstation was used to select the raw anterior projection data and to create ROIs in a manner similar to the one described. The mean MYO:EXT value calculated in stress studies with interference was 1.12 compared to a value of 1.22 in stress studies without interference. Although these values correlated with the qualitative evaluation, in that scans without interference resulted in a higher value, the difference between these values was not statistically significant (*p* = 0.057).

The mean value determined on the rest images with interference was 1.08 and without interference it was 1.24. Here, a strong and statistically significant relationship was determined (*p* = 0.0002). These results suggest that the use of the MYO:EXT in quantifying the amount of interfering extra-cardiac activity might be valuable. However, it seems likely that further confirmatory studies will be needed and that the quest for the reduction of interfering extra-cardiac activity continues.

## Conclusion

The difference between the effect of erythromycin and lemon juice on interfering extra-cardiac activity appears statistically insignificant (*p* = 0.36), and taking into account the better acceptability of erythromycin, this could be considered a suitable alternative to lemon juice. Furthermore, the use of the MYO:EXT ratio seems promising in our setting.
